# Influence of different domains of social capital on psychological distress among Croatian high school students

**DOI:** 10.1186/s13033-015-0010-1

**Published:** 2015-04-02

**Authors:** Dario Novak, Ichiro Kawachi

**Affiliations:** Department of General and Applied Kinesiology, University of Zagreb Faculty of Kinesiology, Zagreb, Croatia; Postdoctoral Fellow at Harvard University, Harvard T.H.Chan School of Public Health, Takemi Program in International Health, Boston, USA; Department of Social and Behavioral Sciences, Harvard T.H.Chan School of Public Health, Boston, Massachusetts USA

**Keywords:** Psychological distress, Social capital, Students, High school

## Abstract

**Background:**

Social capital has been shown to have positive effects on multiple health outcomes among young people (i.e., obesity, diabetes, cardiovascular disease and infectious diseases). Studies are suggesting that social capital is an important asset for the health and wellbeing of children and adolescents, including for their mental health. We sought to examine the influences of different domains of social capital – in the family, in the neighbourhood, and at school – on levels of psychological distress among high school students in Croatia.

**Methods:**

Cross-sectional survey of 3427 high school students (1688 males and 1739 females), aged 17–18 years, was carried out in the 2013/14 school year (response rate: 93.8%). Logistic regression was used to examine the influence of family, neighbourhood and school social capital on the risk of high psychological distress, measured by the Kessler-6 scale.

**Results:**

Adjusting for age, school, gender, body mass index, self-perceived socioeconomic status, self-rated health and physical activity, high family support in school (OR 0.37; 95% CI: 0.27-0.51), high neighbourhood trust (OR 0.62; 95% CI: 0.53-0.73), high teacher-student interpersonal trust (OR 0.74; 95% CI: 0.62-0.89) and high student interpersonal trust (OR 0.79; 95% CI: 0.65-0.97) was each associated with lower odds of psychological distress. When all of the social capital variables were entered simultaneously, higher social capital in each domain was inversely associated with psychological distress.

**Conclusions:**

Family support in school, neighbourhood trust, teacher-student interpersonal trust and student interpersonal trust were significantly inversely associated with psychological distress among adolescents. Intervention and policies that leverage community social capital might serve as means of mental health promotion among youth.

## Background

The prevalence of emotional or behavioural disorders among youth has been rising, and their onset has been pushed earlier with successive birth cohorts. For example, in the U.S., an estimated 20–45% of children and adolescents meet the criteria for emotional and behavioural disorders in the preventing 12 months [1,2]. Croatia is no exception. According to preliminary studies among children and adolescents in Croatia the prevalence rates of behavioural and emotional problems among children/adolescents average around 16.5% [3,4]. Early identification of youth with serious emotional or behavioural problems – including the use of preventive screening - is critical for intervention [5]. It is equally important to identify risk factors and protective factors for the prevention of psychological distress in youth (i.e., risk factors as personal/family injury/illness, parental divorce, conflicting relationship with friends, stress related to academic performance or protective factors as education [1-3,5]).

Recent attention has focused on social capital as a factor contributing to the resilience of children. Social capital is defined as the instrumental and moral resources that individuals can access through their social network connections. In turn, the child is conceptualised as being embedded in social relationships that exist within their families, their neighbourhoods, and their schools [6-9]. Social capital theory posits that interpersonal trust, norms of reciprocity, and exchange of social support each constitutes a type of resource and that access to these resources may promote the resilience of the individuals against adversity [10-12]. Social capital has been shown to have positive effects on multiple health outcomes among young people (i.e., obesity [8], diabetes [9,10], cardiovascular disease [10] and infectious diseases [10]). There are many different layers of environmental influences that can affect a child’s development, starting with people and institutions immediately surrounding the child (i.e., parents and families), to school environments, to residential neighbourhoods, and eventually the societal culture [13]. While an initial paucity of primary research was a constraint [14], the empirical evidence base has accumulated over the last decade with a number of studies suggesting that social capital is an important asset for the health and wellbeing of children and adolescents, including for their mental health [15-20]. Accordingly, we hypothesised that family, neighbourhood and school social capital may be associated adolescent’s high psychological distress and that students who report higher social capital in all three domains will have lower levels of high psychological distress.

In the present study we therefore sought to investigate the influences of different domains of social capital – in the family, in the neighbourhood, and at school – on levels of psychological distress among high school students in Croatia.

## Methods

### Participants

We administered a survey among high school students in Zagreb, a mid-sized urban city in central Croatia with a population of about 1,000,000 people. A random sampling approach was used to select high schools. Twenty schools were selected in Zagreb randomly from a complete list of all private and public schools in the city (out of 86). All of twenty schools that we approached agreed to participate in the survey, representing 3650 students enrolled in the 2013/14 school year. In total 3427 students (1688 males and 1739 females, aged 17–18 years) responded to the survey (93.8% response rate), administered during class. The students ranged in age from 17–18 years (1688 males, body height, 182.11 ± 7.06 cm, body weight, 76.21 ± 10.99 kg, body mass index, 22.95 ± 2.85 kg/m [2] and 1739 females, body height, 168.36 ± 6.41 cm, body weight, 59.07 ± 8.39 kg, body mass index, 20.81 ± 2.54 kg/m [2]). The study was approved by the Faculty of Kinesiology University of Zagreb Institutional Review Board and one of the parents for each subject signed an informed consent form. The students signed an assent form as well.

### Social capital indicators

The survey inquired about perceptions of social capital in the family, neighbourhood, and high school settings [11-13,21]. Family support in school was assessed by the single item: ‘Do you feel your family understands and gives attention to you during high school?’ [11]. Neighbourhood social capital was assessed by two items: ‘Do you feel people trust each other in your neighbourhood (neighbourhood trust)?’ ‘Do you feel that your neighbours step in to criticize deviant behaviour among high school students (informal social control)?’ [11]. School social capital was assessed by three questions; ‘Do you feel teachers and students trust each other in your high school (teacher-student interpersonal trust)?’ ‘Do you feel students trust each other in your high school (student interpersonal trust)?’ ‘Do you feel students collaborate with each another in your high school (students’ collaboration in school)?’ The response options to all questions were on a Likert scale: ‘strongly agree’; ‘agree’; ‘neither agree or disagree’; ‘disagree’; ‘strongly disagree’. The ‘disagree’ and ‘strongly disagree’ responses were combined to create a dichotomous variable indicating lower perceived social capital [11]. The Cronbach alpha of the 3-item school social capital subscale was 0.71. Psychological distress was assessed using the 6-item Kessler scale. Previous studies have shown that the K6 is reliable instrument able to detect mood and anxiety compared to other psychological disorders in adolescents [22]. Each question is scored from 0 (None of the time) to 4 (All of the time). Scores of the 6 questions were then summed (0–24) with lower score indicating low levels of psychological distress. Previous research has shown that the dichotomous scoring of responses in the range 13+ versus 0–12 discriminates accurately between respondents with and without psychological distress, respectively [22,23].

### Covariates

As marker of physical activity, we considered students’ total physical activity in the last 7 days. Physical activity was assessed using the short version of the International Physical Activity Questionnaire (IPAQ) and was expressed as metabolic equivalent-hours per week (MET-hour/week) [24]. Socioeconomic status was entered in our regression models as a potential cofounder, i.e., theoretically associated with both self-rated health and social capital [25]. The classification of SES was based on both parents’ occupations at the time when research was conducted. Self-perceived SES was categorized into three levels: high SES (i.e., managers and professionals), middle SES (white collar) and low SES (blue collar) [26], and was dichotomized as high/middle (responses in the range 2–4) versus low (responses in the range 5–6). Self-rated health was assessed using the standard single item question: “How do you perceive your health?”. Possible responses were arranged along a 5-item Likert type scale: 1 very poor, 2 poor, 3 fair, 4 good, or 5 excellent. Good, very good and excellent were collapsed into one category (good); while fair and poor were designated as poor self-rated health. The measure has also been used previously in adolescents [27-30]. Body mass index (BMI) was used based on self-reported height and weight (≥25 kg/m [2] versus <25 kg/m [2] discriminates between respondents with and without high BMI).

### Data analysis

The association of psychological distress with social capital indicators was examined in four sequential logistic regression models, in which we calculated the odds ratios (ORs) and 95% confidence intervals (CIs) for high psychological distress according to levels of perceived social capital. As potential confounders, we entered age, school, gender, body mass index (BMI), self-perceived socioeconomic status (SES), self-rated health and physical activity. We investigated the association between psychological distress and family support in school (Model 1), neighbourhood social capital (Model 2), and school social capital (Model 3). Finally, we entered all of these social capital variables simultaneously (Model 4). The interaction term between social capital and gender was not statistically significant and shows the results similar for boys and girls so we dropped the sex-stratified analyses. A p-value of <0.05 (two sided) was considered statistically significant. The Statistical Package for the Social Sciences (version 13.0. SPSS, Inc. (2003) was used for data analyses.

## Results

Almost 25% of the participant reported having high psychological distress which is similar to some other surveys [1,31]. The prevalence of psychological distress in girls was twice as high as in boys (33.0% vs. 16.2%). Roughly 20% of the participants reported poor self-rated health (Table 1).

**Table 1:** **Characteristics of the study subjects, Zagreb, Croatia, 2014**

	**Total**	**Males**	**Females**
	**(n = 3427)**	**(n = 1688)**	**(n = 1739)**
Family support in school			
High	3242 (94.60)	1598 (94.66)	1644 (94.54)
Low	185 (5.40)	90 (5.34)	95 (5.46)
Neighborhood trust			
High	2323 (67.78)	1222 (72.39)	1101 (63.31)
Low	1104 (32.22)	466 (27.61)	638 (36.69)
Informal social control			
High	2599 (75.84)	1269 (75.17)	1330 (76.48)
Low	828 (24.16)	419 (24.83)	409 (23.52)
Teacher-student interpersonal trust			
High	2377 (69.36)	1203 (71.27)	1174 (67.51)
Low	1050 (30.64)	485 (28.73)	565 (32.49)
Student interpersonal trust			
High	2587 (75.48)	1349 (79.92)	1238 (71.20)
Low	840 (24.52)	339 (20.08)	501 (28.80)
Students’ collaboration in school			
High	2968 (86.60)	1502 (88.98)	1466 (84.30)
Low	459 (13.40)	186 (11.02)	273 (15.70)
Body mass index			
Normal	3001 (87.56)	1367 (80.98)	1634 (93.96)
Overweight/Obese	426 (12.44)	321 (19.02)	105 (6.04)
Self-rated health			
Good	2763 (80.62)	1449 (85.84)	1314 (75.56)
Poor	664 (19.38)	239 (14.16)	425 (24.44)
Self-perceived socioeconomic status			
High/Middle	2064 (60.22)	1008 (59.71)	1056 (60.72)
Low	1363 (39.78)	680 (40.29)	683 (39.28)
Psychological distress			
High	848 (24.75)	274 (16.24)	574 (33.00)
Low	2579 (75.25)	1414 (83.76)	1165 (67.00)
Physical activity			
High/Moderate	2943 (85.87)	1499 (88.80)	1444 (83.03)
Low	484 (14.13)	189 (11.20)	295 (16.97)

The association between social capital and psychological distress is shown in Table 2. Overall, high psychological distress was significantly inversely associated with each domain of social capital. Adjusted for age, school, gender, BMI, self-perceived SES, self-rated health and physical activity, high family support in school (OR 0.37; 95% CI: 0.27-0.51) and high neighbourhood trust (OR 0.62; 95% CI: 0.53-0.73) were significantly inversely associated with psychological distress. Teacher-student interpersonal trust (OR 0.74; 95% CI: 0.62-0.89) and student interpersonal trust (OR 0.79; 95% CI: 0.65-0.97) were significantly inversely associated with psychological distress. When all of the domains of social capital variables were entered simultaneously in the regression, high family support in school (OR 0.43; 95% CI: 0.31-0.59), high neighbourhood trust (OR 0.69; 95% CI: 0.58-0.82) and teacher-student interpersonal trust (OR 0.80; 95% CI: 0.67-0.96) and student interpersonal trust in school (OR 0.81; 95% CI: 0.66-1.00) each remained significantly inversely associated with psychological distress. Interestingly, our study found that neighbourhood trust was significantly inversely associated with psychological distress whereas informal social control had a slightly adverse impacts on psychological distress, although the estimate was not statistically significant (OR = 1.10, 95% CI: 0.91-1.32).

**Table 2:** **Odds ratios for high psychological distress among high school students, Zagreb, Croatia, 2014**

	**Model 1**	**Model 2**	**Model 3**	**Model 4**
	**OR (95% CI)**	**OR (95% CI)**	**OR (95% CI)**	**OR (95% CI)**
Family support in school				
High	0.37 (0.27-0.51) ***			0.43 (0.31-0.59) ***
Low				
Neighborhood trust				
High		0.62 (0.53-0.73) ***		0.69 (0.58-0.82) ***
Low				
Informal social control				
High		1.07 (0.89-1.28)		1.10 (0.91-1.32)
Low				
Teacher-student interpersonal trust				
High			0.74 (0.62-0.89) ***	0.80 (0.67-0.96) **
Low				
Student interpersonal trust				
High			0.79 (0.65-0.97) *	0.81 (0.66-1.00) *
Low				
Students’ collaboration in school				
High			0.82 (0.64-1.04)	0.84 (0.66-1.07)
Low				
Gender				
Female	2.53 (2.14-2.98) ***	2.40 (2.03-2.83) ***	2.42 (2.05-2.86) ***	2.38 (2.02-2.82) ***
Male				
Body mass index				
Overweight/Obese	1.24 (0.97-1.59)	1.25 (0.98-1.60)	1.28 (1.00-1.63)	1.25 (0.98-1.60)
Normal				
Self-perceived socioeconomic status				
High/Middle	1.00 (0.85-1.18)	1.02 (0.86-1.20)	1.00 (0.84-1.18)	1.01 (0.85-1.19)
Low				
Self-rated health				
Good	0.38 (0.26-0.55) ***	0.38 (0.26-0.55) ***	0.36 (0.26-0.55) ***	0.42 (0.29-0.61) ***
Poor				
Physical activity				
High/Moderate	0.37 (0.26-0.53) ***	0.39 (0.28-0.55) ***	0.40 (0.29-0.57) ***	0.44 (0.31-0.62) ***
Low				

***p<0.001, **p<0.01, *p<0.05; OR – odds ratio; CI – confidence interval.

**Model 1:** Association between family social capital and psychological distress adjusted for age, school, gender, body mass index, self-perceived socioeconomic status, self-rated health and physical activity.

**Model 2:** Association between neighborhood social capital and psychological distress adjusted for age, school, gender, body mass index, self-perceived socioeconomic status, self-rated health and physical activity.

**Model 3:** Association between school social capital and psychological distress adjusted for age, school, gender, body mass index, self-perceived socioeconomic status, self-rated health and physical activity.

**Model 4:** Association between all social capital variables and psychological distress adjusted for age, school, gender, body mass index, self-perceived socioeconomic status, self-rated health and physical activity.

## Discussion

Our findings suggest that young people with higher level of family support in school, higher level of neighbourhood trust, higher level of teacher-student interpersonal trust and higher level of student interpersonal trust are less likely to report psychological distress. Social connectedness within the family, neighbourhood and school thus appear to be important factors influencing mood among young people. Previous studies have suggested that higher levels of family, neighbourhood and school social control were associated with higher levels of children’s and adolescents’ development, mental health and well-being [18,32-35]. Drukker et al. [20] found that mental health and behavioural problems among children and adolescents living in the Netherlands were associated with neighbourhood informal social control, i.e., the supervision of youth by adults in the neighbourhood. Above-mentioned associations were in the same direction, indicating that children living in ‘better’ neighborhoods (low in socio-economic deprivation or high in social capital) had a better mental health and behavior. Aslund et al. [36] showed that low neighbourhood informal social control and low general social trust were associated with higher rates of psychosomatic symptoms, musculoskeletal pain, and depression among Swedish adolescents. Informal social control refers to the role of adults within a community (not just the child’s own parents) in stepping in to prevent the occurrence of ‘deviant’ health behaviours among youth, such as underage smoking and drug abuse.

Our finding of an adverse association between neighbourhood informal control and health was therefore unexpected, and contradicts previous findings reported in the Netherlands and the US [37,38]. The adverse association could have been due to reverse causation, i.e., the possibility that psychological distress could have led to lower assessment of informal social control in the neighborhood. Given the cross-sectional design of our study we cannot exclude the possibility of reverse causation. However, the contradictory findings suggest that the relation between informal social control and health is contingent on the societal and cultural context. In some countries (i.e., Japan), there is a strong norm of preserving social order, which some adolescents find over-powering and stifling. Informal social control is no doubt responsible for the marked absence of graffiti, vandalism, and litter in the streets. However, the strict maintenance of social order may occur at the cost of overly regulating the behaviour of adolescents. In other words, it is “downside” of too strong social capital. There are several identified negative consequences of social capital like exclusion of outsiders, excess claims on group members, restrictions on individual freedom or downward leveling norms (i.e., minorities could be excluded from plush new health services in white neighborhoods or individuals may feel restricted from participating in health promoting activities such as exercise, because of other demanding communal expectancies [10]). We can speculate that for many Croatian teenagers, rebellious acts make sense as a way to express freedom and pull away from the values society attempts to instil in them. To gain freedom in life, some teens’ rebel against the authority figures in their lives [4].

In this study, we have found a statistically significant inverse association between family support in school and psychological distress. For young people, family is important for ‘being there’ in times of need and family members are often regarded as a crucial source of support [39]. Morgan and Haglund reported that the parents’ knowledge about their children’s moods deserves special attention. Parents are the first line of defense in promoting children’s mental health during adversity [40]. Family social capital offers the most consistent protective role for children and adolescents. Parent– child relationships characterized by, for example, positive communication and support were associated with fewer reported mental health and depressive symptoms in the adolescents [41].

We also found that those living in low trust communities reported worse psychological health compared to youth living in high trust communities. As shown in other studies, lack of community support and high stress were associated with greater depressive symptoms [18-20]. Providing safe and enriching opportunities for children and young people to extend and exploit their own social support networks should, therefore, be an important goal for policy makers and practitioners [18,42]. Among the indicators of school social environment, teacher-student interpersonal trust and student interpersonal trust were associated with lower psychological distress, whereas students’ collaboration in school was not significantly associated with psychological distress. A previous study described that these sense of trust between pupils and teachers may promote health by encouraging feelings of safety, acceptance, and support [43].

Our study has some limitations. First, due to the cross-sectional design, we cannot rule out the possibility of reverse causation, i.e., poor mental health led to low level of trust as well as other perceptions of social capital. To mitigate this, we adjusted for self-rated health in order to take account of common source bias, i.e., both the perceptions of social capital as well as psychological distress are subjective and self-reported. This raises the possibility that a third underlying factor (such as self-rated health) could spuriously result in an association between social capital and psychological distress. Second, we used a subjective measure of both health and social capital, so that there is a possibility of common method bias which may have resulted in bias away from the null. Third, because the students completed the surveys during class, there is a possibility of social desirability bias, particularly the assessment of teacher-student interpersonal trust. Fourth, we have used a limited number of items in the measures of social capital and there is no standard/golden pattern tool for the measurement of social capital. Besides, social capital has different domains that could be not contemplated by one of the questions used in the assessment of it in this study. Fifth, the social capital variables in our study are anaylsed at the individual level. Therefore, we are referring to the students’ individual perceptions of social capital. And sixth, all types of social capital were assessed in primary sample. Future studies are warranted to assess all three domains (family, neighbourhood and school social capital) by approaching different sample subjects who are not participating in primary sample.

## Conclusion

In summary, the present study suggests that higher levels of family support in school, neighbourhood trust, teacher-student interpersonal trust and student interpersonal trust were significantly inversely associated with adolescent’s high psychological distress. Interventions are needed like promoting social capital directly by creating new network ties and strengthening students social interaction in all three domains - family, neighbourhood and school. Additional studies are needed to identify those interventions that can increase social capital with the ultimate goal of achieving healthier students.
